# The Radiation Issue in Cardiology: the time for action is now

**DOI:** 10.1186/1476-7120-9-35

**Published:** 2011-11-21

**Authors:** Eugenio Picano, Eliseo Vano

**Affiliations:** 1 Institute of Clinical Physiology, CNR, Pisa, Italy; 2 San Carlos University Hospital, Complutense University of Madrid, Madrid, Spain

**Keywords:** cancer, cardiology, imaging, risk

## Abstract

The "radiation issue" is the need to consider possible deterministic effects (e.g., skin injuries) and long-term cancer risks due to ionizing radiation in the risk-benefit assessment of diagnostic or therapeutic testing. Although there are currently no data showing that high-dose medical studies have actually increased the incidence of cancer, the "linear-no threshold" model in radioprotection assumes that no safe dose exists; all doses add up in determining cancer risks; and the risk increases linearly with increasing radiation dose. The possibility of deterministic effects should also be considered when skin or lens doses may be over the threshold. Cardiologists have a special mission to avoid unjustified or non-optimized use of radiation, since they are responsible for 45% of the entire cumulative effective dose of 3.0 mSv (similar to the radiological risk of 150 chest x-rays) per head per year to the US population from all medical sources except radiotherapy. In addition, interventional cardiologists have an exposure per head per year two to three times higher than that of radiologists. The most active and experienced interventional cardiologists in high volume cath labs have an annual exposure equivalent to around 5 mSv per head and a professional lifetime attributable to excess cancer risk on the order of magnitude of 1 in 100. Cardiologists are the contemporary radiologists but sometimes imperfectly aware of the radiological dose of the examination they prescribe or practice, which can range from the equivalent of 1-60 mSv around a reference dose average of 10-15 mSv for a percutaneous coronary intervention, a cardiac radiofrequency ablation, a multi-detector coronary angiography, or a myocardial perfusion imaging scintigraphy. A good cardiologist cannot be afraid of life-saving radiation, but must be afraid of radiation unawareness and negligence.

## Radiation in cardiology: regulatory framework and missing evidences

Almost 10 years ago, the "radiation issue" was raised, which refers to the need to include long-term cancer risks due to ionizing radiation in the risk-benefit assessment of diagnostic or therapeutic testing. This issue is obviously relevant from the individual patient's [1], societal [2] and bioethical [3] perspective, and clearly stemmed from standard radioprotection knowledge already at that time well-embedded in Euratom law [4] and European Commission medical imaging guidelines [5]. It was initially raised in the critical area of non-invasive diagnosis of coronary artery disease, where the dose of 10 million stress imaging future procedures per year, the high dose of perfusion imaging and the availability of competitive non-ionizing techniques pose special problems of avoidable long-term cancer risk [1,6]. However, at that time this position was largely perceived by peers as being motivated by an attempt of non-radiologist imaging specialists to expand or defend their own imaging market shares [7]. In the last 10 years, things have changed. For a long time ignored by the mainstream imaging and cardiology community, the "linear-no threshold" model in radioprotection assumes that no safe dose exists; the risk increases linearly with increasing radiation dose; all doses add up in determining cancer risk. This model was more generally accepted as epidemiological evidence matured, and was re-endorsed by concordant statements of the US National Academy of Sciences Biological Effects of Ionizing Radiation Committee (2006), International Commission on Radiological Protection (2007), and United Nations Scientific Committee on the Effects of Atomic Energy (2008) [8-10]. Conversely, the hormesis model assuming that low doses of radiation were less harmful and possibly even beneficial was abandoned [8-10] although there are currently no data showing that high dose medical studies have actually increased the incidence of cancer and the full validation of the linear no-threshold model is still lacking in the low dose range (below 100 mSv) [7]. In particular, the evidence gaps are that radiation data gathered from atomic bombings were whole body doses that occurred in a brief period of time, not comparable to small medical doses applied over days or years. Radiation given in fractionated doses as happens with medical testing is probably less harmful than a single dose applied to the same organ. Many of the long-term effects, including cancer, become manifest 20 or more years after the exposure, but diagnostic medical studies are more frequently performed in elderly patients with co-morbidities, less likely to live long enough to develop a radiation-induced illness [7].

In spite of these evidence gaps, in 2005 cardiology imaging guidelines accepted in principle that the risk-benefit assessment balance should include long-term cancer risks on the risk side [11]. In 2005, the interventional cardiology guidelines of the American College of Cardiology Foundation emphasized that "the responsibility of all physicians is to minimize the radiation injury hazard to their patients, to their professional staff and to themselves" [12]. In 2009, the AHA Science Advisory at last delivered the reference doses of common cardiology examinations [13], and in 2010 the ACC committee also overtly expressed the need for appropriate and optimized use of radiation techniques in cardiology [14]. It is now generally recognized that all physicians make every effort to see that "each patient should get the right imaging exam, at the right time, with the right radiation dose", as suggested by the FDA in the 2010 initiative to reduce unnecessary radiation exposure from medical imaging [15]. Attention to radiation protection is one aspect - and not the least important -- of good practice of medicine, and in particular cardiology.

## Cardiologist, the contemporary radiologist

**Figure 1:**
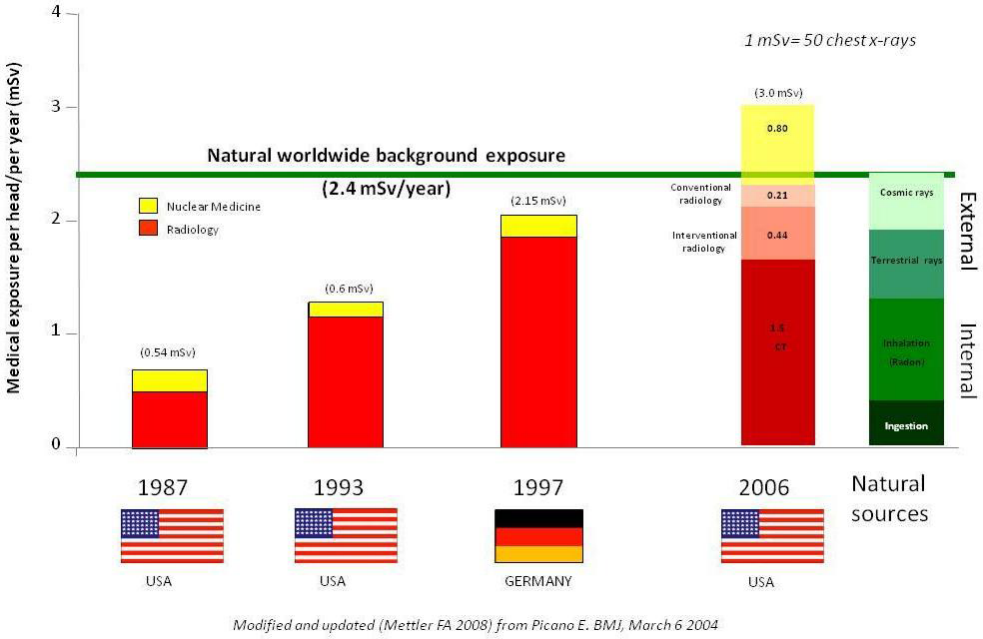
**Medical and natural sources of radiation**. Modified from Picano E, BMJ, 2004, ref. 2 updated with Mettler et al, Health Physics, 2009, ref. 16. The effective dose of 1 mSv is equivalent to 50 chest x-rays.

**Figure 2:**
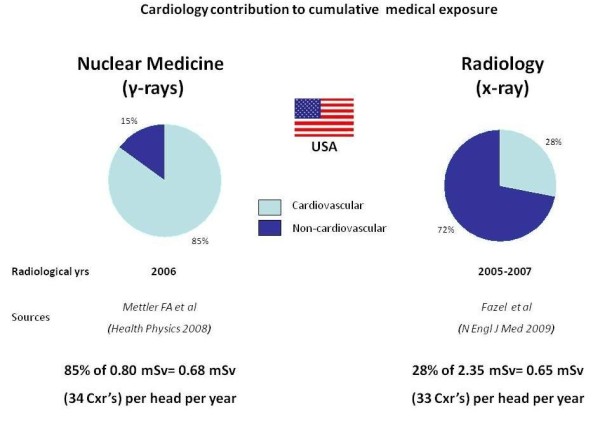
**The relative contribution of cardiovascular examinations to overall exposure from nuclear medicine (left panel) referred to radiological years 2006**. The nuclear cardiology contribution of about 32 chest x-rays per year is matched by the 33 chest x-rays per year from cardiac radiology, for a grand total of 65 chest x-rays, corresponding to 43% of the total exposure of the average US citizen. Redrawn and adapted from ref 16 and 17.

ICRP introduced the quantity effective dose (mSv) for occupational exposures but it is more and more used in medicine as an approach to estimate radiological risk [9]. ICRP recognizes that effective doses can be of value for comparing the relative dose from different diagnostic procedures and for comparing the use of similar technologies and procedures, provided that the referent patient populations are similar with regard to age and sex [9]. Medical radiation from x-rays and nuclear medicine is the largest manmade source of radiation exposure in western countries, accounting for a mean effective dose of 3.0 mSv per head per year, equivalent to the radiological risk of 150 chest x-rays (Figure 1) [16]. Of these, one-fourth come from nuclear medicine (γ-rays) and the remaining from radiology (x-rays). Of the 150 chest x-rays from medical radiation except radiotherapy, almost one-half come from cardiology procedures. In particular, nuclear cardiology accounts for 57% of all nuclear medicine procedures and 85% of the entire cumulative effective dose due to nuclear medicine, whereas cardiac radiology accounts for about 30% of the exposure due to x-ray procedures (Figure 2) [17]. Exact figures can depend upon the specific country, the radiological year, and uncertainties in allocating to a specific subspecialty some examinations, such as chest CT. For instance, in Germany nuclear cardiology accounted for 40% of the overall collective dose from nuclear medicine in the years 1996-2000 [18] and cardiovascular radiology for around 50% of x-rays dose in the radiological year 2005 [19]. Overall there is little doubt that cardiology makes a dominant contribution to global radiological warming. The reasons are simple: 1) cardiology imaging examinations are very common, with about 1 million PCI, 10 million MPI's and 10 million MDCT's per year in the US alone; 2) each procedure involves a very large radiation exposure, which may range from 5 to 57 mSv and more, around an average reference dose of 10-15 mSv for a percutaneous coronary intervention, a cardiac radiofrequency ablation, a multi-detector coronary angiography or a myocardial perfusion imaging scintigraphy [13,20-22]. In particular, effective doses of invasive cardiology procedures vary widely by a factor of 10 (Table 1), with more complex procedures such as dilation of chronic total coronary occlusion [23] or transthoracic aortic valve replacement [24] or endovascular thoraco-abdominal aneurysm repair [25] which may easily exceed the effective dose of 100 mSv. In addition, interventional cardiologists have an exposure per head per year two to three times higher than that of radiologists and their exposure has increased steadily in the past 5 years [26,27]. The most active and experienced of interventional cardiologists in high volume catheterization laboratories have an annual exposure equivalent to around 5 mSv per year, and a professional lifetime attributable excess cancer risk of 1 in 100 [8,28]. For both patients and doctors, the risk is cumulative, meaning that when several test or procedures are performed, dose is added to dose and risk to risk. The cumulative exposure per patient [17,29,30] per problem [31], during a single admission [32] may well reach values around a cumulative exposure of 100 mSv.

**Table 1:** Standard reference doses of common cardiological examinations

**Diagnostic procedures, ref**.	Effective dose (mSv)	Equivalent nmber of PA chest radiographs (each 0.02 mSv)	Approximate equivalent period of natural background radiation (years)
**CONVENTIONAL RADIOGRAPHY**			

Chest x-ray (PA), 13	0.02	1	0.008

**INVASIVE RADIOLOGY**			

Diagnostic coronary angiography, 13	7 (2-16)	350 (100-800)	2.9

PCI, 13	15 ( 7-57)	750 (350-2800)	6.3

Cardiac radiofrequency ablation, 13	15 (7-57)	750 (350-2800)	6.3

Dilation chronic coronary occlusion, 23	81 (17-194)	4050 (850-9600)	33.7

Head and/or neck angiography, 20	5 (1-20)	250	2.1

Thoracic angiography of pulmonary artery or aorta, 20	5 (4-9)	250	2.1

Abdominal angiography or aortography, 20	12 (4-48)	600	5.0

Aortic valvuloplasty, 24	39	1950	16.2

Endovascular thoraco-abdominal aneurysm repair procedures, 24	76-119	3800-5950	31.6-49.5

**COMPUTED TOMOGRAPHY**			

64-slice coronary CTA, 13	15 (3-32)	750 (150-1600)	6.3

Coronary calcium CT,13	3 (1-12)	150 (50-600)	1.2

**NUCLEAR CARDIOLOGY**			

PET F-18 FDG (viability), 13	14	700	5.8

Thallium stress/rest reinjection, 13	41	2050	17

Sestamibi (1 day) stress-rest, 13	9	450	3.7

Rubidium-82, 13	5	250	2.1

N-13 ammonia stress-rest, 22	3	150	1.25

**Figure 3:**
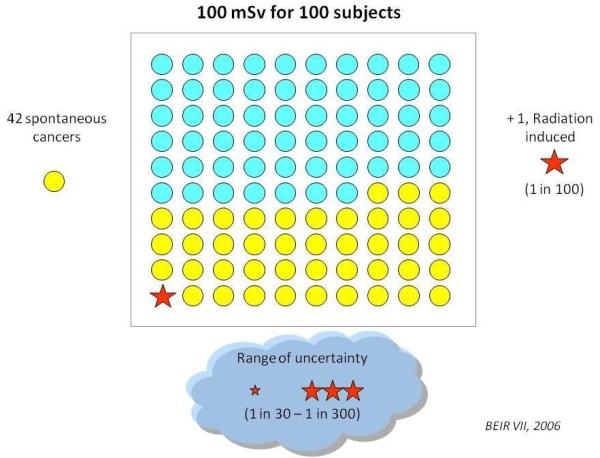
**The risk model of Biological Effects of Ionising Radiation Committee VII for exposure to low-level radiation predicts that about one (red star) out of 100 people would likely develop solid cancer or leukemia from a single exposure of 100 mSv above background**. About 42 additional people (yellow circles) in the same group would be expected to develop solid cancer or leukemia from other causes in a lifetime. Roughly half of these cancers would result in death. Modified and adapted from Committee to Assess Health Risks from Exposure to Low Levels of Ionizing Radiation; Nuclear and Radiation Studies Board, Division on Earth and Life Studies, National Research Council of the National Academies. Health Risks From Exposure to Low Levels of Ionizing Radiation: BEIR VII Phase 2. Washington, DC: The National Academies Press; 2006 (ref. [8]

**A**ccording to current risk estimates if 100 subjects are exposed to 100 mSv, 42 will have a spontaneous cancer (independently of radiation exposure) and 1 will have a radiation-induced cancer (with a range of uncertainty of 1 in 30-1 in 300): Figure 3[8]. This is an average risk, assuming a sex and age distribution similar to that of the entire US population [8]. For any given dose, the risk is 3-4 times higher in children than in adults, 50% lower in an 80-year old compared to a 50-year old subject, and 38% higher in females than in males. These estimates do have a considerable margin of uncertainty, with a 2 to 3 confidence intervals [8].

Dose optimization is essential to minimize both the patient's and doctor's risk in the catheterization laboratory [33]. Decreasing patient dose will result in a proportional decrease in scatter dose to the operator [34]. Therefore, techniques that reduce patient dose will generally also reduce the occupational dose [35]. This is a "win-win" situation: the doctor and the patient both benefit (Table 2). Protective shielding is also essential for operator protection. It includes structural (architectural wall) shielding, mobile shielding (with ceiling suspended leaded plastic and table-suspended drapes) and personal shielding (with lead aprons, thyroid collars and leaded glasses). However, the most effective shielding is the operator's knowledge of radiation risk [36] - which is often suboptimal [37-39].

**Table 2:** Factors modulating doses in cardiac catheterization lab

		Lower doses	Higher doses
**OPERATOR-DEPENDENT**	**Operator background**	**Expert**	**Beginner**

	Cath-lab director	Radiation aware and conscious	Not radiation aware and conscious

	Written records	Includes KAP	Omits KAP

	Arterial Approach	Trans-Femoral	Trans-Radial

	Pulsed Fluoroscopy	Low rate (12.5/s)	High rate (25/s)

	Patient to image intensifier or flat panel distance	As small as possible	Large

	Ventriculography	No	Yes

	Cine-duration	Short	Long

	Magnified views	Few	Many

	Projection	Ant, RAO	Lateral, LAO

	Dose audit	Yes	No

**PATIENT-DEPENDENT**	Body habitus	Lean	Obese

	Coronary lesion to be dilated	Simple and single	Complex and multiple

	Arrhythmic lesion to be ablated	Supraventricular tachycardia	Atrial fibrillation, ventricular tachycardia

**TECHNOLOGY**	X-ray system	Inspected for QC and maintained	Not tested for QC and not maintained

KAP = Kerma Area Product; LAO = Left anterior oblique projection; RAO = Right anterior oblique projection; QC = quality control.

## Tissue reactions in cardiologists and cardiology patients

There are two main biological effects of radiation: stochastic effects, which include carcinogenetic and genetic effects, and tissue reactions (previously called deterministic effects), which cause an immediate and very predictable change to the tissue [40]. Tissue reactions happen when the dose exceeds a specific threshold. The two most frequent examples of tissue reactions (deterministic effects) of cardiological interest are cataract formation (in doctors) and skin injury (in patients).

Cataract, or opacification of the lens, is often associated with visual impairment and may be classified into three main categories: nuclear, cortical, and posterior subcapsular, according to their anatomic location [41]. Of the three major categories of age-related cataracts, posterior subcapsular is the least common but it is the one most frequently associated with ionizing radiation exposure. Because of their location along the lens' visual axis, relatively minor posterior subcapsular cataracts can have great impact on vision. The estimated eye dose is around 0.5 mGy/procedure, in cardiac catheterization laboratories when no eye protection is used. Until recently, the dose threshold for radiation-induced lens opacities were considered 2 Gy for a single dose or 5 Gy for fractionated dose [19]. However, several epidemiological studies among Chernobyl clean-up workers, A-bomb survivors, astronauts, residents of contaminated buildings, and surveys of staff in interventional rooms indicate that there is an increased incidence of lens opacities at doses below 0.5 Gy and even suggests a stochastic hypothesis (non-threshold effect) [42]. Whether deterministic or stochastic in nature, lens opacities have been documented in up to 50% of interventional cardiologists [43]. The reasons for this high prevalence are three fold: first is that operator's eyes are exposed to scattered x-rays; second (avoidable) is the frequent failure of some cardiologists to use protective leaded eyewear [43]; and probably third, that the permitted occupational dose limits were too high even to provoke a mental alert. On April 21, 2011, ICRP slashed the earlier dose limit of 150 mSv in a year for the lens of the eye to the present 20 mSv in a year, averaged over a defined period of five years, with no single year exceeding 50 mSv [44].

When fluoroscopic procedures require more than 20 minutes using high-contrast fluoroscopy mode or 60 minutes in low level fluoroscopy, there may be a possibility of patient skin injuries. Significantly, injuries are not limited solely to the use of older equipment, but can occur when poor technique is employed with newer and digital equipment capable of delivering higher doses [45]. Radiation burns remain asymptomatic and often go unrecognized [46]. This is quite contrary to the familiar thermal burn, which is associated with a recognizable source of heat and instantaneous pain. They usually occur on the patient's back (where the x-rays are delivered) and since they develop several weeks after the procedure their association with cardiac interventions may not be considered, and many severe cases come to light through litigation. A case is filed in US courts every 4-5 weeks by patients who have suffered such injuries [47]. In almost every country around the world, reporting significant radiological incidents and accidents that occur during, or as a direct result of using ionizing radiation for a medical procedure is a legal requirement. But in practice, such reporting hardly ever occurs. Very few countries have a functioning reporting system because no one wants to be blamed for patients' radiation related burns, hair loss or skin injury. However, this information is essential if lessons are to be learned. The IAEA has set up its own international reporting system called SAFRAD (SAFety in RADiological procedures). Because the SAFRAD system is anonymous and the IAEA will not supply identifiable data to governmental authorities or other third parties, there will be no fear of blame [48].

## The future direction of radioprotection in cardiology

We should make every effort to bring the cardiology community from an evidence-poor to an evidence-rich environment in the specific field of radioprotection in cardiology (Table 3). Further data are needed, especially in the low dose range (<100 mSv). BEIR VII listed among top-research needs "future medical imaging studies", including studies of infants who undergo diagnostic exposures [8]. Theoretically, such studies will be a tough challenge, since the extra risk of dying from a single CT scan exposure is estimated to be 1 in 1000, and about 40% of the population eventually have some kind of cancer and 20% of the population will die from it. It has been calculated that an epidemiological study of 5 million people would be required to quantify directly the risk of cancer from exposure of 10 mSv or less [49]. Since the relation linking the required sample size and the exposure dose is hyperbolic, a substantially lower sample size is required if a cumulative diagnostic exposure (of all ionizing tests) is considered (now easily in the range of 100 mSv), if a pediatric or young population is evaluated (in whom fourfold higher effects than an adult are expected for the same radiation dose), and if genetically vulnerable populations are studied (in whom 2 to 3 times the effects of a given dose can be observed in comparison with a genetically resistant population) [50]. Data mining on this very relevant issue has already begun, and in a population of 82,861 patients admitted with acute myocardial infarction, there was a 3% increase in cancer over a mean follow-up of 5 years for every 10 mSv of low-dose ionizing radiation [51]. Another BEIR research need of interest to cardiologists regards "future occupational radiation studies", which can certainly include highly exposed (in the last 2 to 3 decades) interventional cardiologists, a population well suited to assess effects of long-term, low-level radiation exposure in humans [36].

**Table 3:** Action to be taken on radiological protection in cardiology: what can be done

1. Epidemiological data mining in children (especially congenital heart disease with history of intensive interventional procedures)
2. Epidemiological data mining in adults (ischemic heart disease or arrhythmias with history of intensive interventional procedures)

3. Epidemiological data mining in contemporary interventional cardiologists and staff

4. Prospective radiobiology and genetic studies in acutely exposed patients

5. Prospective radiobiology and genetic studies in chronically exposed interventional cardiologist

6. Development of targeted chemo-preventive strategies in high-risk groups (patients and cardiologists)

7. Evaluation of non-cancer (atherosclerosis, reproductive, etc) effects by appropriate biomarkers in exposed staff and patients

8. Development of informatic support to effective dose recording, radiologic risk assessment and imaging appropriateness

9. Development of innovative devices and procedures for radiological protection of patients and doctors

10. Social communication campaign to doctors and patients

Modified and adapted from recommendations of BEIR VII (points 1 to 6); UNSCEAR (point 7); President's Cancer Panel Report (points 8 to 10).

**Figure 4:**
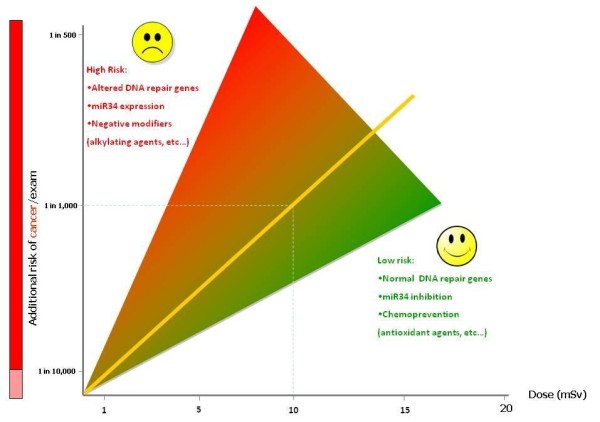
**The population risk is in reality due to the average of a spectrum of risks, with higher risks being for instance associated with mutation of genes involved in DNA repair and with the presence of other environmental mutagens such as smoking**.

A suggested alternative to the epidemiological approach is the biodosimetry approach, applicable to both cardiological patients and professionally exposed cardiologists [4]. The most suitable biodosimeter for cancer is the assay of double-stranded DNA breaks, micronuclei or chromosome aberrations in circulating peripheral lymphocytes, or gamma-H2AX foci for single-stranded DNA damage. In this way, it is easier to "see" in a more tangible way the direct effects of radiation exposure on proximal markers of cancer, which are intermediate end-points and long-term predictors of disease [52,53]. In fact, an acute diagnostic or therapeutic x-ray exposure in the 10- to 50 mSv range - well below the threshold of epidemiological evidence linking radiation to cancer - is associated with a 15% increase in micronuclei in adults after invasive cardiovascular interventions [54], a 100% long-term increase in children treated for congenital heart disease 15 to 20 years after the exposure [55] and a 50% increase in interventional cardiologists after 10 to 20 years of catheterization laboratory exposure with cumulative professional dose in the 30 to 100 mSv range [56]. Chronically exposed interventional cardiologists also show altered redox balance and increased susceptibility to apoptotic induction in lymphocytes [57]. A clear recommendation of UNSCEAR 2009 is to pay more attention "to other non-cancer disease entities, in addition to circulatory diseases", encouraging "future epidemiological studies designed to assess clinical and subclinical endpoints, as well as biomarkers, since this information is more likely to lead to insights" [10]. The challenging field ahead is to translate, for both patients and professionally exposed doctors, the generic population risk obtained from epidemiological age-and gender-based risk into a personalized risk [40]. Several genetic, environmental and dietary variables can affect the variability of damage observed to any given level of radiation, and current research is targeted at shifting epidemiology estimates to personalized measures of DNA and chromosomal damage, focused on identifying inter-individual differences that could modulate radiation risks in order to obtain better estimates of the extent of damage. For instance, radiation-associated chromosomal damage in interventional cardiologists is amplified by smoking and by genetic polymorphism of genes involved in DNA repair [58]. If the risk is personalized, it will be easier to implement targeted predictive and chemo-preventive strategies [59], since it is now proven that a variety of biological response modifiers can modulate tissue reactions in many tissues. These include antioxidants, radical scavengers, anti-inflammatory drugs, angiotensin converting enzyme inhibitors, growth factors and cytokines (Figure 4). In many cases these give dose modifying factors of 1.1 to 2, indicating the potential for increasing threshold doses in known exposure cases. In contrast, there are agents which enhance radiation responses, notably other cytotoxic agents such as antimetabolites, alkylating agents, antiangiogenic drugs, and antibiotics, as well as genetic and comorbidity factors [40].

**Figure 5:**
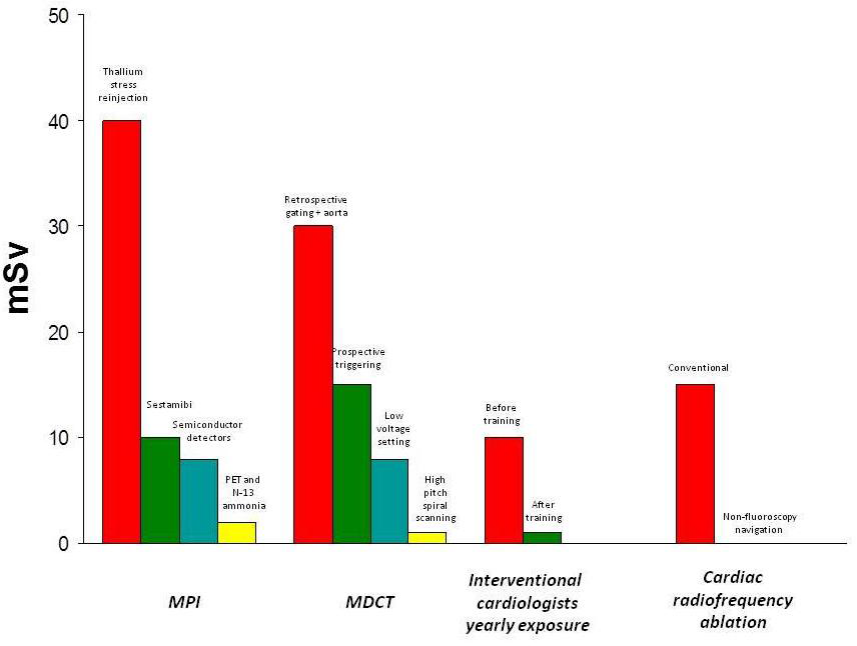
**The radiological dose-sparing cascade provided by technical and cultural upgrading in four critical areas of cardiology**: Myocardial Perfusion Imaging (MPI, from thallium to sestamibi tracers, from standard to triple-headed gamma camera, and from gamma camera to PET imaging with N-13 ammonia), MDCT (Multi-Detector Computed Tomography, from retrospective to prospective triggered techniques with dose modulation), interventional cardiology (with 90% dose reduction simply achieved through radioprotection training) and cardiac radiofrequency ablation (moving from standard fluoroscopy to near-zero exposure with non-fluoroscopy navigation techniques).

Other important cardiology-based lines of research will be technological advancement to reduce the effective dose in different fields, from nuclear cardiology to CT, from interventional cardiology to cardiac radiofrequency ablation (Figure 5). For instance, the recent emphasis on radiation exposure due to CT scanning has engendered a competitive effort on the part of manufacturers (the commercial "dose war", after the "slice war") to reduce the dose while still providing diagnostic images. As a result, cardiac CT angiography can now be performed with high-quality images with a mean effective radiation dose of less than 1 or 2 mSv [60]. Substantial dose reduction can also be achieved in nuclear myocardial perfusion imaging abandoning Thallium (25 mSv+) for Sestamibi (10 mSv), by new reconstruction algorithms, stress-only protocols, and by implementing semiconductor detectors into latest- generation gamma cameras allowing massive scan shortening or dose reduction [22]. Research is currently addressing the issue of finding a way to improve the unacceptably high rate of inappropriate cardiac imaging testing [61], still around 30% for most common ionizing examinations such as myocardial perfusion scintigraphy or cardiac CT [62,63]. Last but not least, substantial effort will be directed to increasing the currently suboptimal radiological awareness of cardiologists, prescribing doctors and patients, since awareness is the best shield from unnecessary medical radiation exposure. Both FDA [15] and the President's Cancer Panel [64] recommend social communication to patients and doctors. The pattern of this communication might be modelled on the "Image Gently and Step Lightly Campaign", which in the US addressed the issue of radiological responsibility, focusing on the risks of unnecessary and excessive medical radiation from interventional radiology administered to our pediatric patients [65]. User-friendly informatic support or mobile platforms might be helpful for this purpose [66]. Another highly effective, and possibly the best, way to improve the radiological awareness within the cardiology community is to involve cardiologists in a proactive role on studies evaluating the health effects of radiation on themselves. The Multispecialty Occupational Health Group (MOHG) undertook a cohort mortality study comparing cancer and other serious disease outcomes (including cardiovascular diseases and cataracts) in 44,000 physicians performing fluoroscopically guided procedures (including interventional cardiologists, radiologists, neuroradiologists and others) and in 12,000 non-interventional radiologists with risks in 101,000 physicians who are unlikely to be occupationally exposed to radiation (e.g., family physicians or psychiatrists) [67]. Member organizations of the MSOHG include the Society of Cardiac Angiography and Intervention, Society of Interventional Radiology, Heart Rhythm Society, American College of Radiology, American College of Cardiology, Society of Neurointervention Surgery, American Association of Physicists in Medicine, and Society of Invasive Cardiac Professionals. The MSOHG is collaborating with experts in occupational health, epidemiology, and radiation effects from the United States Navy and the Radiation Epidemiology Branch of the National Cancer Institute, to perform epidemiological studies addressing the fundamental questions important to all those working in such an environment.

In Italy, the Healthy Cath Lab study is organized by the Italian National Research Council with endorsement of Italian Society of Invasive Cardiologists, and is designed by interventional cardiologists on interventional cardiologists and for interventional cardiologists (http://www.gise.it.healthycathlab). The Italian study population will consist of 500 exposed (high, medium, and low exposure) interventional cardiologists and staff (technicians and staff) and 500 unexposed controls (clinical cardiologists and nurses). With this limited sample size, the detection of potentially increased health risks remains difficult through the epidemiological approach. Therefore, as an alternative to the epidemiological approach the Healthy Cath Lab study will assess non-cancer health effects through "early warning signs", which evaluate initial damage through surrogate endpoints which are easy to measure, non-invasive, and are able to identify long-term risk for subsequent clinically overt disease, such as micronuclei as a surrogate for cancer, telomere length for atherosclerosis and aging, and so on [53].

## Risk estimates: uncertainties and controversies

Cardiologists have to rely on the best available estimates of risk, and at present such evidence is represented by the linear-no-threshold model presented by BEIR VII [8]. Other organizations are basically supportive of this model, including the International Commission on Radiological Protection (2007) [9,40], United Nations Scientific Committee on the Effects of Atomic Radiation (2008) [10], National US Council of Radiation Protection and Measurements (2001) [68] and UK National Radiological Protection Board (1995) [69]. Other organizations such as the Health Physics Society believe (2004) that LNT is an oversimplification and risk estimates should not be used at < 50 mSv [70]. The French Academy of Sciences (2004) and American Nuclear Society (2001) hold that LNT overestimates risk [71,72].

**Figure 6:**
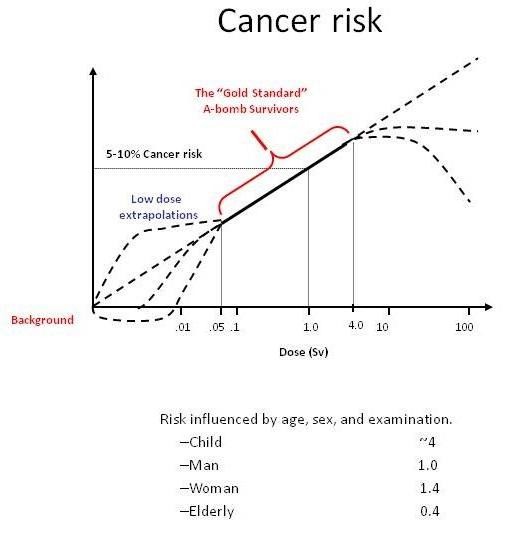
**The dose-effect relationship between radiation exposure and cancer**. The solid line indicates the epidemiological evidence, which is conclusive for doses above 50 to 100 mSv. The dashed line indicates the dose range with absent or inconclusive evidence.

It is our opinion that physicians cannot enter into the radiobiological dispute and should simply accept and apply in their daily practice the LNT model and BEIR risk estimates which are incorporated into law in many countries. The BEIR VII risk models were developed based on a comprehensive review of the world literature on radiation epidemiology, and extensive efforts were made to compose a highly expert committee and avoid conflicts of interest. The conclusion is that the LNT best fits the data and should remain the standard for radiation protection although still suffering from substantial indetermination in the low dose range (Figure 6). The risk of cancer also evaluated by BEIR VII (or ICRP) needs to be weighed against the potential benefits of any radiation-based diagnostic or therapeutic procedure [73]. In addition, the lively debate on the existence of a threshold < 50-100 mSv is de facto outdated by the high levels of dose exposure in contemporary patients, who easily cross the threshold in one episode of care, or with a single exam [29-32]. A more substantial uncertainty relates to the entity of cancer risk for any given dose, since both BEIR VII and ICRP apply a reduced estimate of cancer risk based on risk coefficients derived from the Japanese atomic bomb survivors, that is, from persons with acute, high-dose exposures. Such acute, high dose estimates are then combined with a "dose and dose-rate effectiveness factor" (DDREF). Values for this correction factor have mainly been deduced from experiments with laboratory animals and from radiobiological measurements. Specifically, the ICRP-derived estimates of the excess cancer risk after low-dose exposures and after exposures with higher doses but low-dose rates by reducing the corresponding risk value for the atomic bomb survivors by a DDREF of 2.0 [9]. The BEIR VII Committee of the US National Research Council used a DDERF of 1.5 [8]. In the last 10 years, 12 epidemiological studies on cancer after low-dose rate, moderate-dose exposures were included in the analysis of cancer risk related to such exposures [74], and the excess relative risk per dose values were greater than those published by BEIR VII and ICRP. In addition, the possibility of non-cancer health effects currently not accepted in the radioprotection regulating framework are considered increasingly likely, for instance for atherosclerotic effects, proven on epidemiological grounds for doses higher than 500 mSv [40]. According to ICRP, a dose of 500 mSv may lead to approximately 1% of exposed individuals developing cardiovascular or cerebrovascular disease, more than 10 years after the exposure, in addition to the 30-50% suffering from disease independently of the exposure [40]. Cardiologists, researchers and scientific societies should make every effort to move from the current evidence-poor to an evidence-rich milieu, with data directly linking radiation exposure to cancer and non-cancer effects in our patients and in ourselves as exposed population. In the meantime, the adoption of BEIR VII or ICRP estimates - a prudent trade-off between scientific evidence and judgement, and therefore more likely to fall on the conservative side of risk estimate -- is recommended.

## Conclusions

In the recent past, sometimes cardiologists have been unaware of the radiological dose of the examination they prescribe or practice, but they should make every effort to reduce unnecessary radiation exposure from medical imaging. This is best obtained through a systematic implementation of the 3A's strategy proposed by the International Atomic Energy Agency in 2011: Audit (of true delivered dose); Appropriateness (since at least one-third of examinations are inappropriate); Awareness (since the knowledge of doses and risks is still largely suboptimal in doctors and patients) [75]. It can be repeated for imaging and invasive cardiologists what has been recently written of radiologists: "they must walk a digital tightrope strung between too much and too little radiation. they must image gently, but not too gently - striking a balance between patient risk and diagnostic value" [76]. The recognition of risks inherent in the use of a known carcinogen such as radiation also opens unprecedented new opportunities for scientific [77], social, technological, bioethics [78,79] and medical advancement, of interest to scientists, clinical cardiologists, patients, and industry [80]. A good cardiologist - and even more so, a good imaging or interventional cardiologist - cannot be afraid of radiation, but must be very afraid of radiation unawareness.

## Competing interests

The authors declare that they have no competing interests.

## Authors' contributions

EP drafted the manuscript. EV critically reviewed it, and also wrote parts related to dosimetry. All authors read and approved the final manuscript.

## References

[B1] Picano E Stress echocardiography: a historical perspective Am J Med 2003 114 126 30 Special Article 10.1016/S0002-9343(02)01427-4 12586232

[B2] Picano E Sustainability of medical imaging BMJ 2004 328 578 80 Education and Debate 10.1136/bmj.328.7439.578 15001510 PMC381057

[B3] Picano E Informed consent and communication of risk from radiological and nuclear medicine examinations: how to escape from a communication inferno BMJ 2004 329 849 851 Education and debate 10.1136/bmj.329.7470.849 15472270 PMC521582

[B4] Council Directive 97/43/Euratom of 30 June 1997 on health protection of individuals against the dangers of ionising radiation in relation to medical exposure, and repealing Directive 84/466/Euratom Official Journal of the European Communities 1997 L 180 0022 7

[B5] European Commission on Radiation Protection 118: Referral guidelines for imaging http://ec.europa.eu/energy/nuclear/radioprotection/publication/doc/118_en.pdf (last accessed September 21, 2011)

[B6] Berrington de Gonzalez A Kim KP Smith-Bindman R McAreavey D Myocardial perfusion scans: projected population cancer risks from current levels of use in the United States Circulation 2010 122 2403 10 10.1161/CIRCULATIONAHA.110.941625 21098448 PMC3548424

[B7] Abbott BG Zaret BL Contemporary cardiology and hysteric nucleophobia Am J Med 2003 114 131 4 10.1016/S0002-9343(02)01523-1 12586233

[B8] Committee to Assess Health Risks from Exposure to Low Levels of Ionizing Radiation. Health risks from exposure to low levels of ionizing radiation: BEIR VII Phase 2. Washington, DC: The National Academies Press; 2006 http://www.nap.edu/openbook.php?isbn=030909156X Available at: Last accessed September 21, 2011 25077203

[B9] The 2007 Recommendations of the International Commission on Radiological Protection. Publication 103 Ann ICRP 2007 37 1 332 18082557 10.1016/j.icrp.2007.10.003

[B10] UNSCEAR 2008 Report "Sources and effects of ionizing radiation" I

[B11] Brindis RG Douglas PS Hendel RC Peterson ED Wolk MJ Allen JM Patel MR Raskin IE Hendel RC Bateman TM Cerqueira MD Gibbons RJ Gillam LD Gillespie JA Hendel RC Iskandrian AE Jerome SD Krumholz HM Messer JV Spertus JA Stowers SA American College of Cardiology Foundation Quality Strategic Directions Committee Appropriateness Criteria Working Group American Society of Nuclear Cardiology American Heart Association ACCF/ASNC appropriateness criteria for single-photon emission computed tomography myocardial perfusion imaging (SPECT MPI): a report of the American College of Cardiology Foundation Quality Strategic Directions Committee Appropriateness Criteria Working Group and the American Society of Nuclear Cardiology endorsed by the American Heart Association J Am Coll Cardiol 2005 46 1587 605 10.1016/j.jacc.2005.08.029 16226194

[B12] Hirshfeld JW Jr Balter S Brinker JA Kern MJ Klein LW Lindsay BD Tommaso CL Tracy CM Wagner LK ACCF/AHA/HRS/SCAI Clinical Competence Statement on Physician Knowledge to Optimize Patient Safety and Image Quality in Fluoroscopically Guided Invasive Cardiovascular Procedures: A Report of the American College of Cardiology Foundation/American Heart Association/American College of Physicians Task Force on Clinical Competence and Training Circulation 2005 111 511 532 10.1161/01.CIR.0000157946.29224.5D 15687141

[B13] Gerber TC Carr JJ Arai AE Dixon RL Ferrari VA Gomes AS Heller GV McCollough CH McNitt-Gray MF Mettler FA Mieres JH Morin RL Yester MV Ionizing radiation in cardiac imaging: a science advisory from the American Heart Association Committee on Cardiac Imaging of the Council on Clinical Cardiology and Committee on Cardiovascular Imaging and Intervention of the Council on Cardiovascular Radiology and Intervention Circulation 2009 119 1056 65 10.1161/CIRCULATIONAHA.108.191650 19188512

[B14] Brindis R Douglas PS President's page: The ACC encourages multi-pronged approach to radiation safety J Am Coll Cardiol 2010 56 522 4 10.1016/j.jacc.2010.07.001 20670765

[B15] Food and Drug Administration White Paper: Initiative to Reduce Unnecessary Radiation Exposure from Medical Imaging. Initiative to reduce unnecessary radiation exposure http://www.fda.gov/Radiation-EmittingProducts/RadiationSafety/RadiationDoseReduction/ucm199994.htm]

[B16] Mettler FA Jr Bhargavan M Faulkner K Gilley DB Gray JE Ibbott GS Lipoti JA Mahesh M McCrohan JL Stabin MG Thomadsen BR Yoshizumi TT Radiologic and nuclear medicine studies in the United States and worldwide: frequency, radiation dose, and comparison with other radiation sources--1950-2007 Radiology 2009 253 520 31 10.1148/radiol.2532082010 19789227

[B17] Fazel R Krumholz HM Wang Y Ross JS Chen J Ting HH Shah ND Nasir K Einstein AJ Nallamothu BK Exposure to low-dose ionizing radiation from medical imaging procedures N Engl J Med 2009 361 849 57 10.1056/NEJMoa0901249 19710483 PMC3707303

[B18] Hacker M Schnell-Inderst P Nosske D Weiss M Stamm-Meyer A Brix G Hahn K Radiation exposure of patients undergoing nuclear medicine procedures in Germany between 1996 and 2000. Multicenter evaluation of age and gender-specific patient data Nuklearmedizin 2005 44 119 30 16163407 10.1267/nukl05040119

[B19] Regulla DF Eder H Patient exposure in medical X-ray imaging in Europe Radiat Prot Dosimetry 2005 114 11 25 10.1093/rpd/nch538 15933076

[B20] Mettler FA Jr Huda W Yoshizumi TT Mahesh M Effective doses in radiology and diagnostic nuclear medicine: a catalog Radiology 2008 248 254 63 10.1148/radiol.2481071451 18566177

[B21] Einstein AJ Moser KW Thompson RC Cerqueira MD Henzlova MJ Radiation dose to patients from cardiac diagnostic imaging Circulation 2007 116 1290 305 10.1161/CIRCULATIONAHA.107.688101 17846343

[B22] Kaufmann PA Knuuti J Ionizing radiation risks of cardiac imaging: estimates of the immeasurable Eur Heart J 2011 32 269 71 10.1093/eurheartj/ehq298 20829211

[B23] Suzuki S Furui S Issiki T Kozuma K Koyama Y Yamamoto H Ochai M Asakima Y Ikari Y Patients' skin dose during percutaneous intervention for chronic total occlusion Cath Cardiov Interv 2008 71 160 64 10.1002/ccd.21284 17932885

[B24] Panuccio G Greenberg RK Wunderle K Mastracci TM Eagleton MG Davros W Comparison of indirect radiation dose estimates with directly measured radiation dose for patients and operators during complex endovascular procedures J Vasc Surg 2011 53 885 894.e1 discussion 894 10.1016/j.jvs.2010.10.106 21292431

[B25] Signorotto P Del Vecchio A Montorfano M Maisano F Giagnorio M Bellanca R Colombo A Calandrino R Dosimetric data and radiation risk analysis for new procedures in interventional cardiology Radiat Prot Dosimetry 2010 in press 10.1093/rpd/ncq208 20858680

[B26] Miller DL Vañó E Bartal G Balter S Dixon R Padovani R Schueler B Cardella JF de Baère T Cardiovascular and Interventional Radiology Society of Europe Society of Interventional Radiology Occupational radiation protection in interventional radiology: a joint guideline of the Cardiovascular and Interventional Radiology Society of Europe and the Society of Interventional Radiology Cardiovasc Intervent Radiol 2010 33 230 9 10.1007/s00270-009-9756-7 20020300 PMC2841268

[B27] Vano E Radiation exposure to cardiologists: how it could be reduced Heart 2003 89 1123 4 10.1136/heart.89.10.1123 12975391 PMC1767905

[B28] Venneri L Rossi F Botto N Andreassi MG Salcone N Emad A Lazzeri M Gori C Vano E Picano E Cancer risk from professional exposure in staff working in cardiac catheterization laboratory: insights from the National Research Council's Biological Effects of Ionizing Radiation VII Report Am Heart J 2009 157 118 24 10.1016/j.ahj.2008.08.009 19081407

[B29] Bedetti G Botto N Andreassi MG Traino C Vano E Picano E Cumulative patient effective dose in cardiology Br J Radiol 2008 81 699 705 10.1259/bjr/29507259 18508874

[B30] Ait-Ali L Andreassi MG Foffa I Spadoni I Vano E Picano E Cumulative patient effective dose and acute radiation-induced chromosomal DNA damage in children with congenital heart disease Heart 2010 96 269 74 10.1136/hrt.2008.160309 19687017

[B31] Einstein AJ Weiner SD Bernheim A Kulon M Bokhari S Johnson LL Moses JW Balter S Multiple testing, cumulative radiation dose, and clinical indications in patients undergoing myocardial perfusion imaging JAMA 2010 304 2137 44 10.1001/jama.2010.1664 21078807 PMC3667407

[B32] Kaul P Medvedev S Hohmann SF Douglas P Peterson ED Patel MR Ionizing radiation exposure to patients admitted with acute myocardial infarction in the United States Circulation 2010 122 2160 69 10.1161/CIRCULATIONAHA.110.973339 21060076

[B33] Kuon E Radiation exposure in invasive cardiology Heart 2008 94 667 74 10.1136/hrt.2007.125021 18411362

[B34] Padovani R Le Heron J Cruz-Suarez R Duran A Lefaure C Miller DL Sim HK Vano E Rehani M Czarwinski R International project on individual monitoring and radiation exposure levels in interventional cardiology Radiat Prot Dosimetry 2011 144 437 41 10.1093/rpd/ncq326 21051431

[B35] Klein LW Miller DL Balter S Laskey W Haines D Norbash A Mauro MA Goldstein JA Joint Inter-Society Task Force on Occupational Hazards in the Interventional Laboratory Occupational health hazards in the interventional laboratory: time for a safer environment J Vasc Interv Radiol 2009 20 S278 83 10.1016/j.jvir.2009.04.027 19560009

[B36] Vaño E Gonzalez L Fernandez JM Alfonso F Macaya C Occupational radiation doses in interventional cardiology: a 15-year follow-up Br J Radiol 2006 79 383 8 10.1259/bjr/26829723 16632618

[B37] Watson RM Radiation exposure: clueless in the cath lab, or sayonara ALARA Cathet Cardiovasc Diagn 1997 42 126 7 10.1002/(SICI)1097-0304(199710)42:2<126::AID-CCD5>3.0.CO;2-F 9328691

[B38] Correia MJ Hellies A Andreassi MG Ghelarducci B Picano E Lack of radiological awareness among physicians working in a tertiary-care cardiological centre Int J Cardiol 2005 103 307 10.1016/j.ijcard.2004.08.070 16098394

[B39] Kim C Vasaiwala S Haque F Pratap K Vidovich MI Radiation safety among cardiology fellows Am J Cardiol 2010 106 125 8 10.1016/j.amjcard.2010.02.026 20609659

[B40] Annals of the ICRP Early and late effects of radiation in normal tissues and organs: threshold doses for tissue reactions and other non-cancer effects of radiation in a radiation protection context Draft report for consultation ICRP ref 4834-1783-0153 2011 2 10.1016/j.icrp.2012.02.001 22925378

[B41] Rehani MM Vano E Ciraj-Bjelac O Kleiman NJ Radiation and cataract Radiat Prot Dosimetry 2011 10.1093/rpd/ncr299 21764807

[B42] Vano E Kleiman NJ Duran A Rehani MM Echeverri D Cabrera M Radiation cataract risk in interventional cardiology personnel Radiat Res 2010 174 490 495 10.1667/RR2207.1 20726724

[B43] Ciraj-Bjelac O Rehani MM Sim KH Liew HB Vano E Kleiman NJ Risk for radiationinduced cataract for staff in interventional cardiology: is there reason for concern? Catheter Cardiovasc Interv 2010 76 826 834 10.1002/ccd.22670 20549683

[B44] ICRP Statement on Tissue Reactions. Approved by the Commission on April 21, 2011 http://www.icrp.org/docs/ICRP%20Statement%20on%20Tissue%20Reactions

[B45] Balter S Hopewell JW Miller DL Wagner LK Zelefsky MJ Fluoroscopically guided interventional procedures: a review of radiation effects on patients' skin and hair Radiology 2010 254 326 41 10.1148/radiol.2542082312 20093507

[B46] Vlietstra RE Wagner LK X-ray burns--painful, protracted, and preventable Clin Cardiol 2008 31 145 7 10.1002/clc.20204 18404681 PMC6653362

[B47] Rehani MM Srimahachota S Skin injuries in interventional procedures Radiation Protection Dosimetry 2011 1 5 10.1093/rpd/ncr257 21737442

[B48] SAFRAD - SAFety in RADiological procedures http://rpop.iaea.org/safrad

[B49] Land CE Estimating cancer risks from low doses of ionizing radiation Science 1980 209 1197 203 10.1126/science.7403879 7403879

[B50] Brenner DJ Doll R Goodhead DT Hall EJ Land CE Little JB Lubin JH Preston DL Preston RJ Puskin JS Ron E Sachs RK Samet JM Setlow RB Zaider M Cancer risks attributable to low doses of ionizing radiation: assessing what we really know Proc Natl Acad Sci USA 2003 100 1376 6 10.1073/pnas.2235592100 PMC283495 14610281

[B51] Eisenberg MJ Afilalo J Lawler PR Abrahamowicz M Richard H Pilote L Cancer risk related to low-dose ionizing radiation from cardiac imaging in patients after acute myocardial infarction CMAJ 2011 183 430 6 10.1503/cmaj.100463 21324846 PMC3050947

[B52] Beels L Bacher K De Wolf D Werbrouck J Thierens H Gamma-H2AX foci as a biomarker for patient X-ray exposure in pediatric cardiac catheterization: are we underestimating radiation risks? Circulation 2009 120 1903 9 10.1161/CIRCULATIONAHA.109.880385 19858412

[B53] Vasan RS Biomarkers of cardiovascular disease. Molecular basis and practical considerations Circulation 2010 113 2335 62 10.1161/CIRCULATIONAHA.104.482570 16702488

[B54] Andreassi MG Cioppa A Manfredi S Palmieri C Botto N Picano E Acute chromosomal DNA damage in human lymphocytes after radiation exposure in invasive cardiovascular procedures Eur Heart J 2007 28 2195 9 10.1093/eurheartj/ehm225 17598926

[B55] Andreassi MG Ait-Ali L Botto N Manfredi S Mottola G Picano E Cardiac catheterization and long-term chromosomal damage in children with congenital heart disease Eur Heart J 2006 27 2703 8 10.1093/eurheartj/ehl014 16717079

[B56] Andreassi MG Cioppa A Botto N Joksic G Manfredi S Federici C Ostojic M Rubino P Picano E Somatic DNA damage in interventional cardiologists: a case-control study FASEB J 2005 19 998 9 15802491 10.1096/fj.04-3287fje

[B57] Russo GL Tedesco I Russo M Cioppa A Andreassi MG Picano E Cellular adaptation to chronic radiation exposure in interventional cardiologists Eur Heart J 2011 10.1093/eurheartj/ehr263 21862464

[B58] Andreassi MG Foffa I Manfredi S Botto N Cioppa A Picano E Genetic polymorphisms in XRCC1, OGG1, APE1 and XRCC3 DNA repair genes, ionizing radiation exposure and chromosomal DNA damage in interventional cardiologists Mutat Res 2009 666 57 63 10.1016/j.mrfmmm.2009.04.003 19393248

[B59] Andreassi MG Cioppa A Manfredi S Neri MG Foffa I Picano E N-acetyl cysteine reduces chromosomal DNA damage in circulating lymphocytes during cardiac catheterization procedures: A pilot study Int J Cardiol 2011 10.1016/j.ijcard.2011.05.001 21605919

[B60] Gahadri JR Küest SM Goetti R Image quality and radiation dose comparison of prospectively triggered low-dose CCTA: 128-slice dual-source high-pitch spiral versus 64-slice single-source sequential acquisition Int J Cardiovasc Imaging 2011 10.1007/s10554-011-9921-3 21744246

[B61] Picano E Pasanisi E Brown J Marwick TH A gatekeeper for the gatekeeper: inappropriate referrals to stress echocardiography Am Heart J 2007 154 285 90 10.1016/j.ahj.2007.04.032 17643578

[B62] Gibbons RJ Miller TD Hodge D Urban L Araoz PA Pellikka P McCully RB Application of appropriateness criteria to stress single-photon emission computed tomography sestamibi studies and stress echocardiograms in an academic medical center J Am Coll Cardiol 2008 51 1283 9 10.1016/j.jacc.2007.10.064 18371560

[B63] Ayyad AE Cole J Syed A Desai MY Halliburton S Schoenhagen P Flamm SD Sola S Temporal trends in utilization of cardiac computed tomography J Cardiovasc Comput Tomogr 2009 3 16 21 10.1016/j.jcct.2008.10.009 19201372

[B64] President's Cancer Panel: Environmentally caused cancers are "grossly underestimated" and "needlessly devastate American lives" http://www.environmentalhealthnews.org/ehs/news/presidents-cancer-panel

[B65] Sidhu M Coley BD Goske MJ Connolly B Racadio J Yoshizumi TT Utley T Strauss KJ Image Gently, Step Lightly: increasing radiation dose awareness in pediatric interventional radiology Pediatr Radiol 2009 39 1135 8 10.1007/s00247-009-1392-5 19693493

[B66] Carpeggiani C Paterni M Caramella D Vano E Semelka R Picano E A novel tool for user-friendly estimation of natural, diagnostic and professional radiation risk: Radio-Risk software Eur J Radiology 2011 in press 10.1016/j.ejrad.2011.05.039 21820256

[B67] Linet MS Kim KP Miller DL Kleinerman RA Simon SL Berrington de Gonzalez A Historical review of occupational exposures and cancer risks in medical radiation workers Radiat Res 2010 174 793 808 10.1667/RR2014.1 21128805 PMC4098897

[B68] Upton AC Adelstein SJ Brenner DJ Report No. 136--Evaluation of the Linear-Nonthreshold Dose-Response Model for Ionizing Radiation Bethesda, MD: National Council on Radiation Protection and Measurements (NCRP) 2001

[B69] Cox R Muirhead CR Stather JW Edwards AA Little MP Risk of radiation-induced cancer at low doses and low dose rates for radiation protection purposes Documents of the NRPB 1995 6 1 77

[B70] Health Physics Society. Radiation risk in perspective: Position statement of the Health Physics Society 2004 http://hps.org/documents/radiationrisk.pdf Adopted January 1996, revised Accessed June 14, 2007 10.1097/HP.0000000000001157 31703015

[B71] Tubiana M Aurengo A Averbeck D Dose-effect relationships and estimation of the carcinogenic effects of low doses of ionizing radiation 2005 http://www.academie-medecine.fr/upload/base//rapports_227_fichier_lie.rtf Accessed June 14, 2007 10.1016/j.ijrobp.2005.06.013 16168825

[B72] American Nuclear Society Health effects of low-level radiation: position statement 2001 http://www.ans.org/pi/ps/docs/ps41.pdf Accessed June 14, 2007

[B73] Einstein AJ Henzlova MJ Rajagopalan S Estimating risk of cancer associated with radiation exposure from 64-slice computed tomography coronary angiography JAMA 2007 298 317 23 10.1001/jama.298.3.317 17635892

[B74] Jacob P Rühm W Walsh L Blettner M Hammer G Zeeb H Is cancer risk of radiation workers larger than expected? Occup Environ Med 2009 66 789 96 10.1136/oem.2008.043265 19570756 PMC2776242

[B75] Malone J Guleria R Craven C Horton P Järvinen H Mayo J O'reilly G Picano E Remedios D Leheron J Rehani M Holmberg O Czarwinski R Justification of diagnostic medical exposures, some practical issues: report of an International Atomic Energy Agency Consultation Br J Radiol 2011 10.1259/bjr/42893576 PMC3479887 21343316

[B76] Freihen GT New technologies promise dramatic cuts in CT dose 2011 Medscape cardiology news. Medpulse newsletter

[B77] Gori T Münzel T Biological effects of low-dose radiation: of harm and hormesis Eur Heart J 2011 Editorial 10.1093/eurheartj/ehr288 21862465

[B78] Picano E Matucci-Cerinic M Unnecessary radiation exposure from medical imaging in rheumatology patients Rheumatology 2011 50 1537 39 10.1093/rheumatology/keq412 21183451

[B79] Baerlocher MO Detski AS Discussing radiation risks associated with CT scans with patients JAMA 2010 304 2170 2171 10.1001/jama.2010.1591 21081730

[B80] Limacher MC Zaher CA Walsh MN Wolf WJ Douglas PS Schwartz JB Wright JS Bodycombe DP The ACC professional life survey: career decisions of women and men in cardiology. A report of the Committee on Women in Cardiology. American College of Cardiology J Am Coll Cardiol 1998 32 827 35 10.1016/S0735-1097(98)00319-2 9741533

